# Research trends and hotspots of recurrent pregnancy loss with thrombophilia: a bibliometric analysis

**DOI:** 10.1186/s12884-022-05210-z

**Published:** 2022-12-16

**Authors:** Ying-jun Deng, Sheng-Jing Liu, Ming Zhao, Feng Zhao, Jun Guo, Yu-xiao Huang

**Affiliations:** 1 Department of Andrology, Xiyuan Hospital of China Academy of Chinese Medical Sciences, Beijing, 100091 China; 2 Department of Obstetrics and Gynecology, Xiyuan Hospital of China Academy of Chinese Medical Sciences, No.1 Xiyuan Cao Chang, Zhongzhi Road, Beijing, 100091 China

**Keywords:** Thrombophilia, Recurrent pregnancy loss, Bibliometric analysis

## Abstract

**Background:**

Thrombophilia is a group of disorders that result in a blood hypercoagulable state and induce thrombosis, which was found widely existed in recurrent pregnancy loss (RPL). More and more research about thrombophilia has been conducted but the association between thrombophilia and RPL remains uncertain. Thus, it’s necessary to combine relevant literature to find the research hotspots and analyze the internal link between different study points, and then predict the development trend in RPL with thrombophilia.

**Methods:**

Relevant articles between 1970 and 2022 were obtained from the Web of Science (WoS) database. Software VOSviewer and CiteSpace were used to perform the analysis and conduct visualization of scientific productivity and emerging trends.

**Results:**

Seven hundred twenty-five articles published in recent 30 years by 3205 authors from 1139 organizations and 68 countries were analyzed. 37authors, 38 countries, and 53 organizations published papers ≥5. The United States was the most productive country and Univ Amsterdam was the most productive institution. Journal *thrombosis and haemostasis* had the most total citations. In keyword and clusters, factor-v-Leiden, inherited thrombophilia, activated protein-c, low-dose aspirin, molecular-weigh heparin, polymorphism had high-frequency focus on its etiology, diagnostics, and therapeutics. The strongest keyword bursts showed the research hotspots changed over time.

**Conclusions:**

There could be differences in the clinical relevance of different type of thrombophilia, as well as single and multiple thrombophilic factors. Anticoagulation and immunotherapy are currently the main treatment options. More clinical trials and basic research are expected and we should attach more attention to the whole management of in-vitro fertilization in the future.

**Supplementary Information:**

The online version contains supplementary material available at 10.1186/s12884-022-05210-z.

## Introduction

The hemostatic system adjust into a hypercoagulable state in women during pregnancy was originally a means of protection, but can predispose both the mother and fetus to complications such as pre-eclampsia, placental abruption, fetal growth restriction (FGR), and recurrent pregnancy loss (RPL) [1]. This hypercoagulable state that leads to complications in pregnancy is thought to be associated with a propensity for thrombosis, as well as thrombophilia, which is a group of disorders that result in a blood hypercoagulable state and induce thrombosis [2, 3]. In recent years, the relationship between thrombophilia and RPL is attracting more and more attention in medical research. One study showed that at least one thrombophilic defect was found in most patients with RPL [4]. And a hypothetical mechanism is that the pregnancy loss caused by thrombosis in decidual vessels, impairing the blood supply to the fetus and thus leading to fetal death [5]. Low molecular heparin and some immunological agents are used in the treatment of this disease in order to obtain a pregnancy outcome with live birth [6–8]. Large scale of research are conducted in thrombophilia with RPL, involving diagnostic markers, mechanisms, and treatments [9–11]. Due to the lack of similar systematic literature studies and reviews at present, therefore, it is necessary to combine relevant literature to find the research hotspots and the internal link between different study, and then predict the development trend in RPL with thrombophilia, thus providing some reference for the study of RPL.

Bibliometric Analysis is quantitative tools commonly used in scientific research, provides a model for quantitative analysis of scientific literature to understand the development process and cutting-edge trends in the research field through a large number of literature studies [12, 13]. Aimed to systematically analyze the current status of research in this field, identify research hotspots and limitations of current research, and predict its development trends, We undertook a comprehensive scientometrics review of the development of the RPL with thrombophilia research. Softwares of CiteSpace, VOSviewer, Endnote,and Microsoft Excel were used to analyze countries/regions, institutions, authors, co-citation and keywords based on bibliometric analysis [14–16]. We use figures and tables to show the development process and the current status of research in this area. The results of these analyses (1) reveal the document pattern of RPL with thrombophilia research at the global scale;(2) look at the changes in research hotspots over the past 30 years;(3) discover the frontier areas and trends of the discipline;(4) identify gaps in current research and strategies in future.

## Materials and methods

### Data source and search strategy

The ISI Web of Science (WoS) core database was selected as the data source, as Web of Science was the only database available to track citation counts and organizes a very comprehensive set of articles with enough data to support a bibliometric analysis. It allows analysis of bibliometric indicators relating to researchers, institutions, countries and regions [17, 18]. The literature search was conducted on February 15, 2022 and covered the period from 1970 to 2022. We limited literature types to “article”, and the language of publications were English. We search terms with various combinations of the following terms: “recurrent miscarriage”, “recurrent Abortions”, “habitual abortion”, “recurrent pregnancy loss”, with “thrombophilia”, the following search string was used in this study [19, 20]. (TS = recurrent pregnancy loss and TS = thrombophilia; TS = recurrent miscarriage and TS = thrombophilia; TS = recurrent Abortions and TS = thrombophilia; TS = habitual abortion and TS = thrombophilia).

Finally, 1347 publication records were exported with each record containing author, title, source.

document, abstract, and cited references. All eligible data from the WoS were downloaded in txt and endnote files for further analysis. After exporting, we merged multiple exported files to the same txt file.

### Literature inclusion and exclusion

Then use Citespace 5.1 software to remove the duplicates. After that, screening research papers by reading abstracts. As far as possible, we adopted all articles with both RPL and thrombophilia topics, while removing articles with other diseases as topics under thrombophilia topics, such as thrombophilia and stroke, heart disease, etc. All article types were preserved except abstracts. The literature was initially screened through the titles and abstracts to eliminate literature that was not relevant to the RPL with thrombophilia study. Two investigators independently performed the inclusion and exclusion analysis of the literature and checked the evaluation results with each other, and negotiated to resolve any disagreement or asked a third party to make a determination (The flow diagram of literature screening process is presented in Appendix 1). The processed txt file was used for software analysis of the Bibliometric Analysis.

### Data processing

In order to get more valuable visual analysis results, we further processed the text. Firstly, keywords related to the topic words, such as thrombophilia, recurrent pregnancy loss, etc., were removed, and those without substantial meaning (such as review, study, etc.) were removed. We then use the VOSviewer thesaurus to unify the different expressions of the same object into a unique form. The keywords with the same meaning were combined, such as factor-v-leiden and factor v leiden. The author named such as “goddijn, mariette” and “goddijn, m”. And similar cases in region/country, constitutions and others.

### Statistical analysis

Data mining, analysis and visualization were conducted by VOSviewer (version 1.6.16), CiteSpace (5.0.1), and Microsoft Excel (2019). These software are used individually or in combination in bibliometric studies in various natural science fields, such as agricultural, clinical medicine, pharmacology and so on [16, 21, 22].

VOSviewer (1.6.16) was developed by VanEck and Waltman at the Centre for Science and Technology Studies (CWTS), Leiden University, the Netherlands, in 2009, is mainly oriented toward documentary data, relational knowledge units of documents construction [16]. It is adapted to the analysis of one-mode undirected networks and focus on the visualization of scientific knowledge. It was used to identify keywords, author keywords, productive countries, organizations, and the main co-cited journals, related visual networks were also constructed. CiteSpace (5.0. R1) he software is citation visualization and analysis software gradually developed in the context of scientometrics and data visualization, was used to construct cluster analysis of high-frequency keywords based on the clustering function and annual burst keywords. We managed the data and analyzed the publication tendency using Microsoft Office Excel 2019 (Microsoft Corporation, Redmond, WA, United States). In the VOSviewer network maps, the size of the nodes reflects the number of studies or co-occurrence frequencies, the links between nodes represent the co-occurrence relationships, and the size of the links indicates the cooccurrence frequencies of two nodes. The impact factors (IFs) of the academic journals were collected from the 2022 Journal Citation Reports (JCR) (Clarivate Analytics, Philadelphia, United States).

We also used Endnote (version 20.0.1) software for literature management. The advantage of Endnote is that it can manage all fields of literature and perform classified retrieval, which can help us locate specific documents based on the macro analysis of bibliometrics, so as to conduct more accurate analysis [23].

## Results

### Analysis on the amount of published papers

**Fig. 1:**
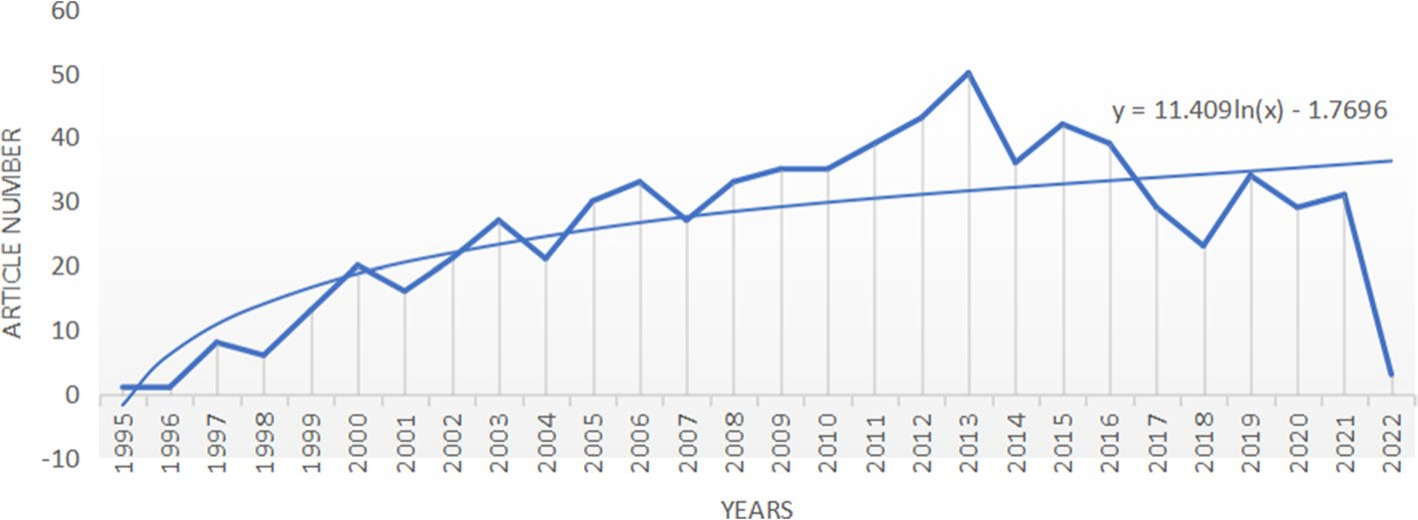
Numbers of articles by year of publication related to Thrombophilia from 1995 to 2022

Finally, 725 papers remained after weeded by reading the title and abstract. We queried the database to aggregate papers by the year of publication. As we can see in Fig. 1, the first article about thrombophilia and RPL was published in 1995. In 2013, the number of publications reached a peak, which may be related to some landmark events and requires continued analysis based on other results. Until now the number of published articles maintained an average annual publication volume of 30 globally. We experimented with different regression models and found the logistic regression model that provided the best fit for data. Using this, we derived the forecast equation as y = 11.409ln(x)-1.7696. Using the equation, we hypothesize that the annual number of publications in the next few years may also be around 30.

### Countries, institutes and authors

We listed the rank of country and institution by the number of documents and citations to see the industry power and internal relationship among them. The top 10 most contributed countries and institutes about thrombophilia and RPL were shown in Tables 1 and 2. In terms of number of publish articles, The United States was the top1 of all the countries and the Univ Amsterdam was the top1 in all the institutions. That may be related to the high total number of research institutions about this topic in the United States, with strong links to individual countries. The Univ Amsterdam, on the other hand, has a clear advantage in terms of the number of articles published by its individual institutions.

**Table 1:** The top 10 countries and institutes contributed to publications about thrombophilia with RPL

Rank	Country	Document	Citations	Institution/Country	Documents	Citations
1	The United States	103	3632	Univ Amsterdam/ Netherlands	28	1531
	Germany	70	1755	Rambam Med Ctr/Israel	25	1413
3	England	66	3496	Tel Aviv Univ/Israel	15	982
4	Israel	66	3392	Leiden Univ/Netherland	15	654
5	Italy	64	2652	Technion Israel Inst /Israel	13	556
6	Netherlands	48	2384	Univ Ottawa Hosp/Canada	11	490
7	France	43	1697	King Edward Mem Hosp/India	10	137
8	Turkey	38	297	Chaim Sheba Med Ctr/Israel	9	683
9	Spain	32	1466	Univ Foggia/Italy	9	228
10	China	31	260	Univ Belgrade/Serbia	9	31

**Table 2:** Author contributed to publications about thrombophilia with RPL

Rank	Author	Organization	Documents	Citations
1	Benjamin Brenner	Rambam Med Ctr/Israel	26	1548
2	Saskia Middeldorp	Univ Amsterdam /Netherlands	24	922
3	Mariëtte Goddijn	Univ Amsterdam/Netherlands	15	582
4	Elvira Grandone	Casa Sollievo Della Sofferenza/Italy	13	460
5	Toth Bettina	Heidelberg Univ/Germany	11	263
6	Younis, JS	Poriya Hosp/ Israel	10	999
7	L Regan	Univ London Imperial Coll/England	10	963
8	Gris, Jean-Christophe	CHU de Nimes/France	10	504
9	Glueck, Charles J.	Jewish Hosp Cincinnati/United States	9	324
10	Zeev Blumenfeld	Rambam Med Ctr/Israel	8	781

**Fig. 2:**
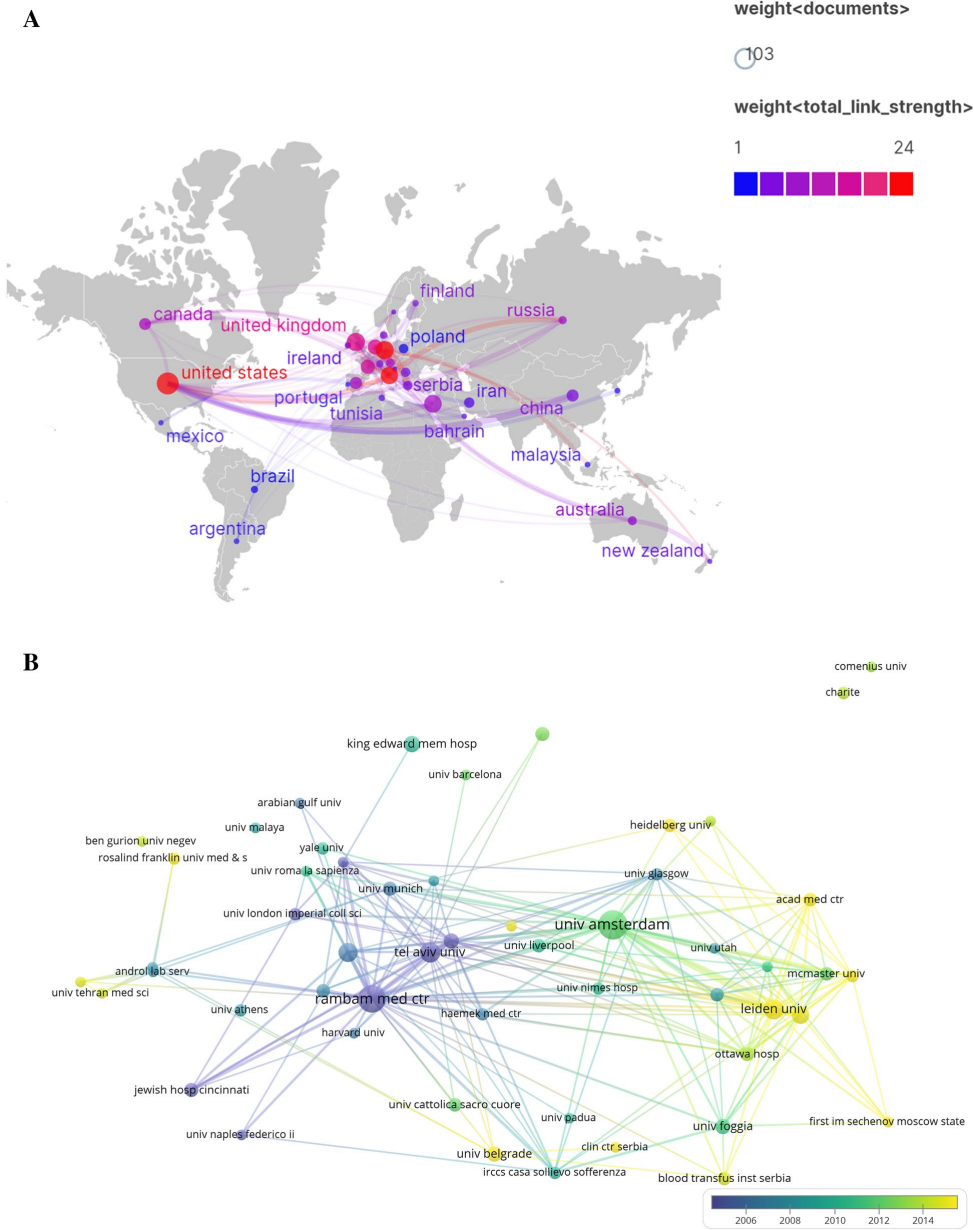
The distribution of countries, institutions, authors, and journals on thrombophilia and RPL research. **a** Map of countries with publications on thrombophilia and RPL. **b** Map of institutions with publications on thrombophilia and RPL

**Fig. 3:**
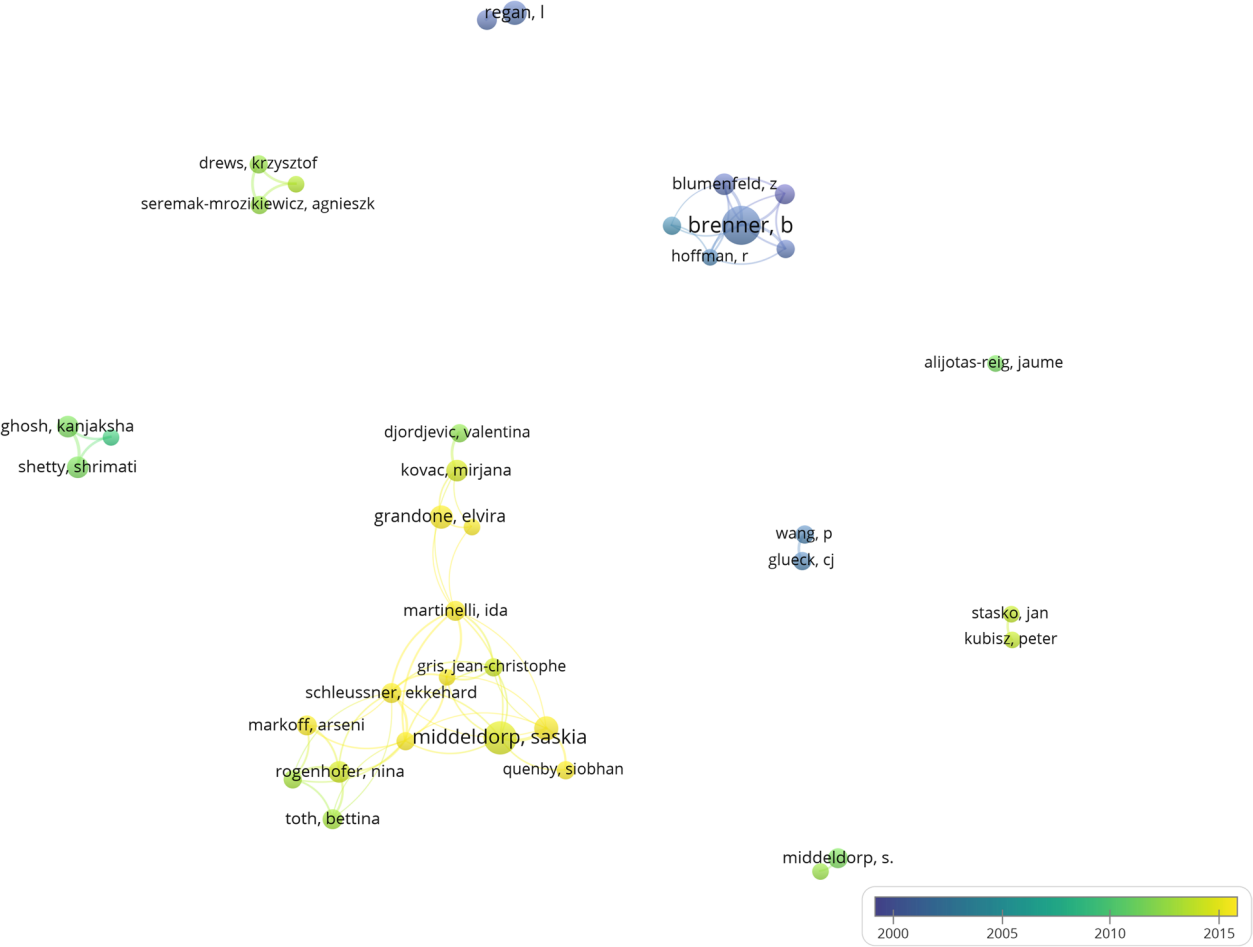
Map of authors with publications on thrombophilia and RPL

Figure 2 shows the connections between institutes, and countries. The sizes of nodes reflected the publications quantity and the width of lines reflects the collaborations between them in the figure. we reserved the documents ≥5 in the Fig.2a and b, 38 countries and 53 institutions achieve the requirement. Based on Fig.2a and b, we further searched and sorted all the articles with multi-country participation by retrieving the author address field of Endnote, found that studies with multinational co-participation were generally multicenter case-control studies or cohort studies. Such as the research about causal link between heritable thrombophilia and fetal loss [24], and studies of the effect of anticoagulants on pregnancy outcome in women with thrombophilia [25, 26]. Probably due to the workload and the difficulty of implementation, the number of articles inter-country collaborations is rare, but the impact of the articles were significant [27]. The institutions that cooperate more with foreign countries are also institutions with outstanding research in this field domestic, like Univ Amsterdam in Netherlands, Rambam Med Ctr in Israel, Leiden Univ in Holland and so on. We can see such research teams in Fig. 3. Thirty-seven authors’ published papers ≥5 of the all 3205 authors in adopted articles (1.15%). Different research teams have some similar research topics such as the casual link between inherited thrombophilia and RPL, and clinical application of anticoagulants in RPL, as we can see in Benjamin Brenner team, from Rambam Med Ctr in Israel, and Saskia Middeldorp team, from Univ Amsterdam in Netherlands [28–30]. No significant exchange between academic groups was found, except European countries.

### Papers and journals

**Fig. 4:**
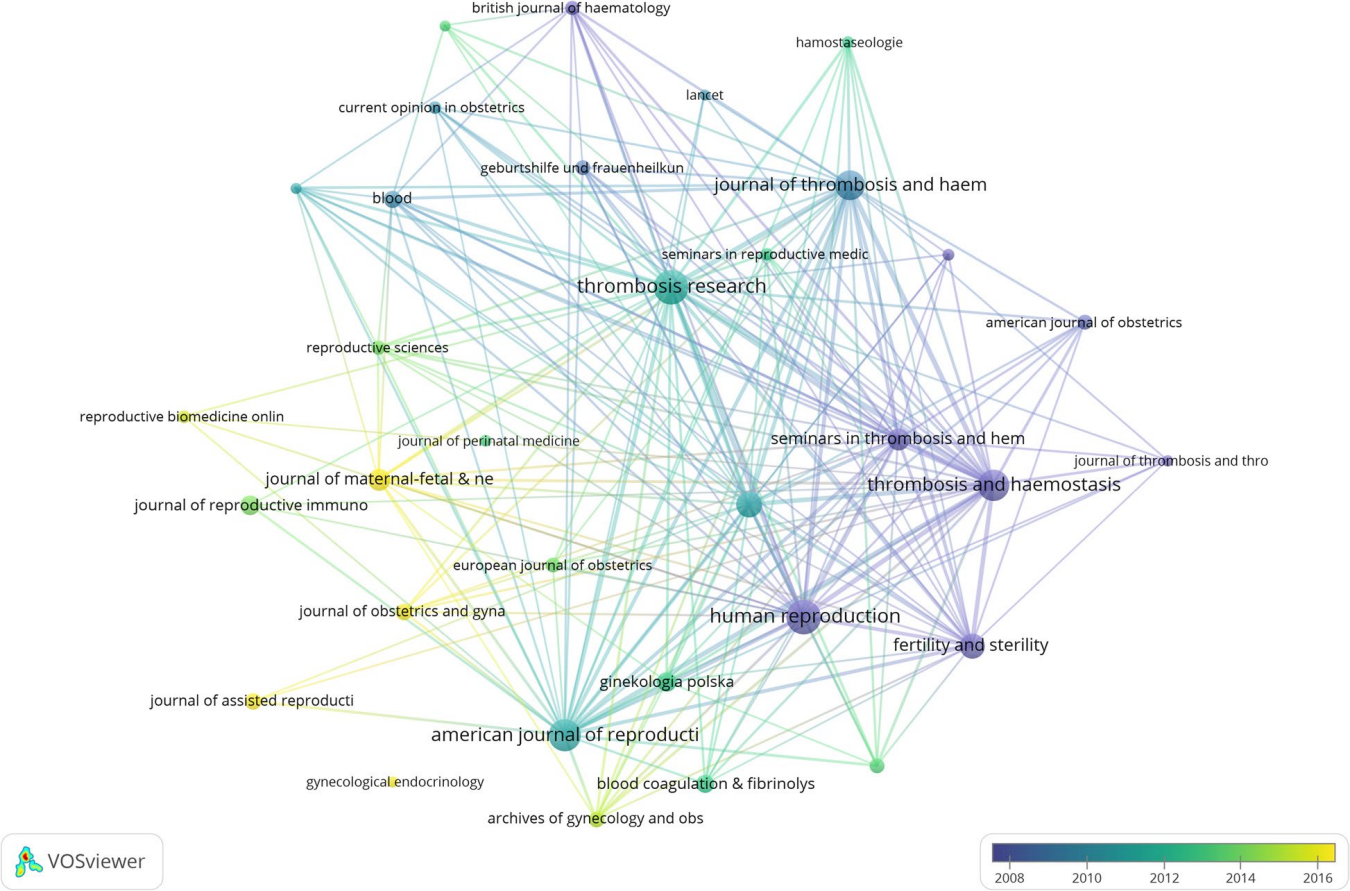
Map of journals with publications on thrombophilia and RPL

All the 725 articles were published in 226 journals, set Boundary value as 5,33 journals reserved in Fig. 4. The top 10 co-cited journals and articles about thrombophilia with RPL were listed in Tables 3 and 4. *Thrombosis and haemostasis* had the most total citations, and the most cited article was Rey (2003) “Thrombophilic disorders and fetal loss: a meta-analysis” published on *Lancet* [19]. Most high cited papers were published before 2010 may means Major groundbreaking work was completed by 2010 in this area. The most recent article was published in 2012 titled “Guidelines on the investigation and management of antiphospholipid syndrome” [31]. This may be related to the spike in postings that occurred in 2013, and until now, antiphospholipid syndrome still is a hot topic in the study of RPL. Due to several high quality randomized controlled clinical trials and cohort studies in multiple centers, *Lancet* has a very high citation rate with a small number of documents. How thrombophilia leads to RPL is still a critical question and the diagnosis and treatment of it is still ongoing in the world.

**Table 3:** The top 10 co-cited journals about thrombophilia with RPL

Rank	Journal	IF	Country	Documents	Citations
1	Thrombosis and Haemostasis	5.249	Germany	33	2219
2	Human Reproduction	6.918	England	39	1617
3	Lancet	60.39	England	5	1375
4	American journal of Reproductive Immunology	3.886	United States	35	1128
5	Fertility and Sterility	7.329	United States	23	859
6	Journal of Thrombosis and Haemostasis	5.824	England	31	797
7	Blood	17.543	United States	12	577
8	British Journal of Haematology	5.518	England	8	563
9	Seminars in Thrombosis and Hemostasis	2.892	United States	18	451
10	Thrombosis Research	2.869	United States	40	447

**Table 4:** The top 10 highly cited articles about thrombophilia with RPL

Rank	Document	Title	Journal	Citations
1	Rey [19]	Thrombophilic disorders and fetal loss: a meta-analysis	Lancet	585
3	Regan (2000)	Recurrent miscarriage - an aspirin a day?	Human Reproduction	322
4	Kaandorp (2010)	Aspirin plus Heparin or Aspirin Alone in Women with Recurrent Miscarriage	New England Journal of Medicine	289
5	Keeling [31]	Guidelines on the investigation and management of antiphospholipid syndrome	British Journal of Haematology	278
6	Haverkate (1995)	Familial dysfibrinogenemia and thrombophilia. Report on a study of the SSC Subcommittee on Fibrinogen	Thromb Haemost	264
7	Martinelli (2000)	Mutations in coagulation factors in women with unexplained late fetal loss	New England Journal of Medicine	253
8	Brenner [29]	Gestational outcome in thrombophilic women with recurrent pregnancy loss treated by enoxaparin	Thrombosis and Haemostasis	239
9	Laskin (2009)	Low Molecular Weight Heparin and Aspirin for Recurrent Pregnancy Loss: Results from the Randomized, Controlled HepASA Trial	Journal of Rheumatology	232
10	grandone (1997)	Factor V Leiden is associated with repeated and recurrent unexplained fetal losses	Thrombosis and Haemostasis	231

### Keywords and clusters

**Fig. 5:**
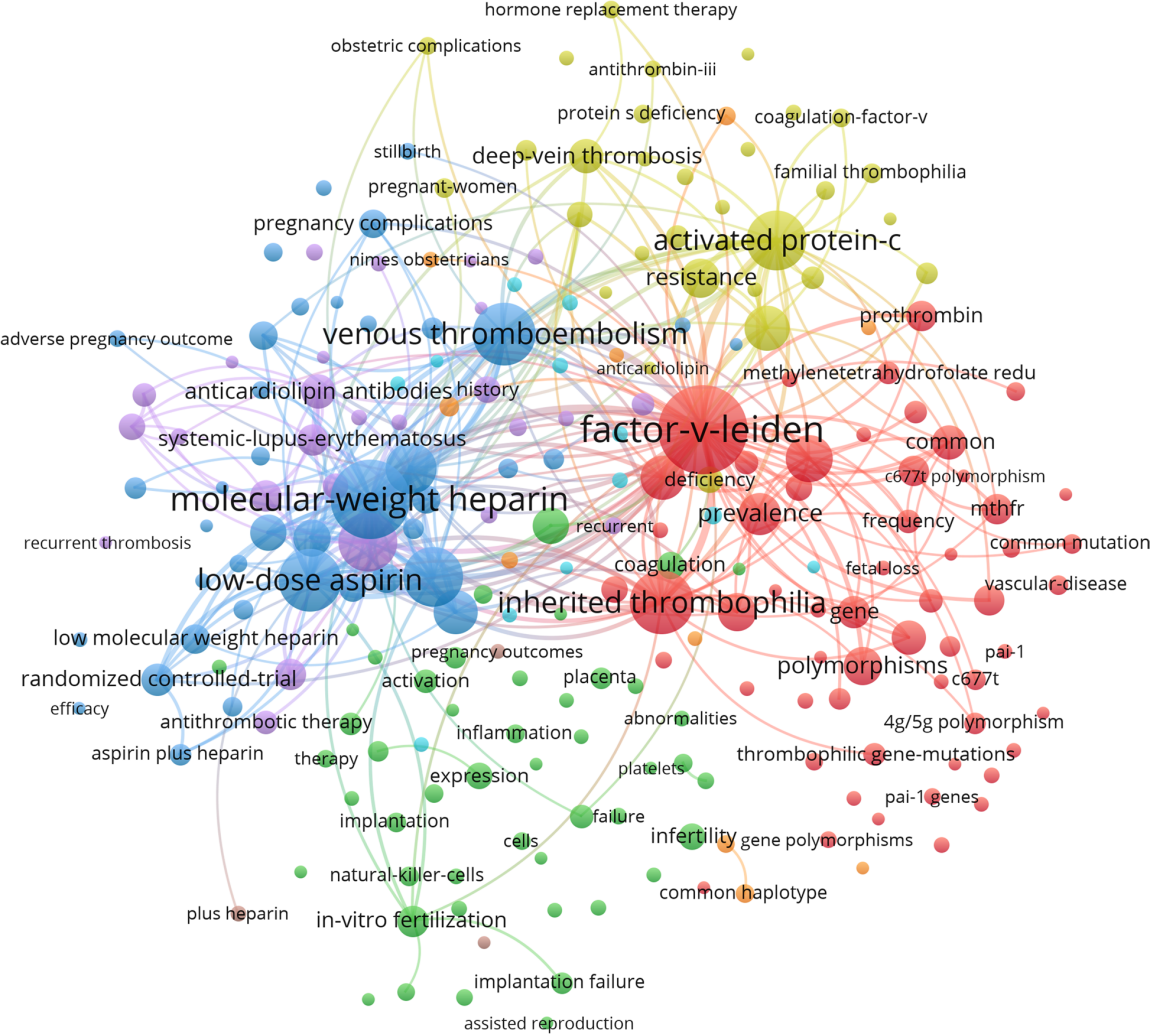
The co-occurrence analysis of keywords on thrombophilia with RPL

There were 2096 keywords in 725 articles that were analyzed. For a better understanding of the relationship among them, the keywords meaning to correlative subject headings were removed from the list, and the keywords with similar meanings were merged for analysis. The frequencies of keywords greater than or equal to 5 (T ≥ 5) were used to construct the co-occurrence network map, as shown in Fig. 5. (The nodes size reflects the frequency of keywords, and the lines between nodes reflects the correlation between keywords.). The significant keywords include factor-v-Leiden, inherited thrombophilia, activated protein-c, low-dose aspirin, molecular-weigh heparin, polymorphism, etc. All the keywords indicated that research about thrombophilia and RPL focused on its etiology, diagnostics, and therapeutics.

**Fig. 6:**
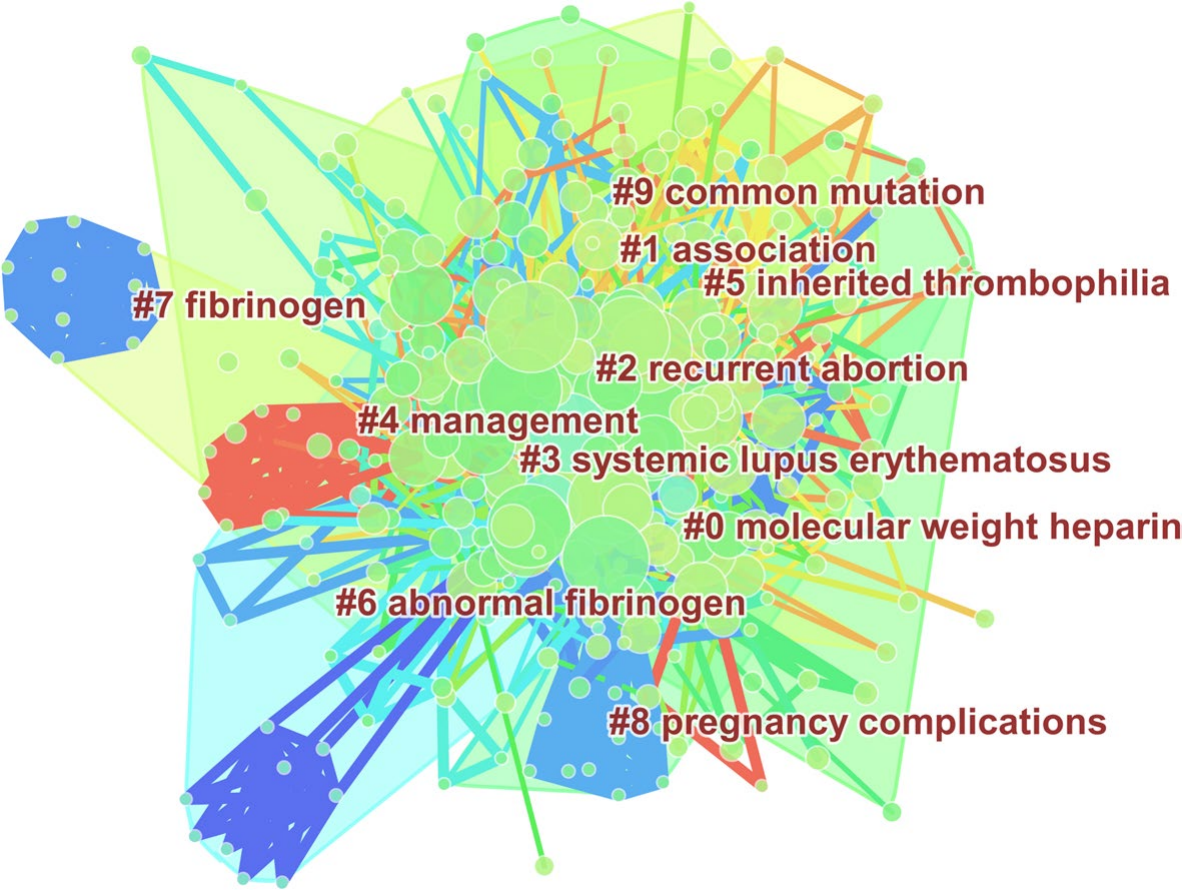
The cluster map of keywords on thrombophilia with RPL

Cluster analysis using the K-means algorithm was conducted and identified 10 distinct clusters as shown in Fig. 6. The Q value was 0.43 (Q value is the clustering module value, Q > 0.3 means that the clustering result is significant), and the S value was 0.64 (s value is the average contour value of clustering, s > 0.5 means that the clustering is reasonable). Cluster1, 5, and 9 showed the association between inherited thrombophilia and RPL. Cluster 3, 6, and 7 showed acquired thrombophilia with RPL. Cluster 0 emphasized the application of anticoagulants in thrombophilia in RPL.

**Fig. 7:**
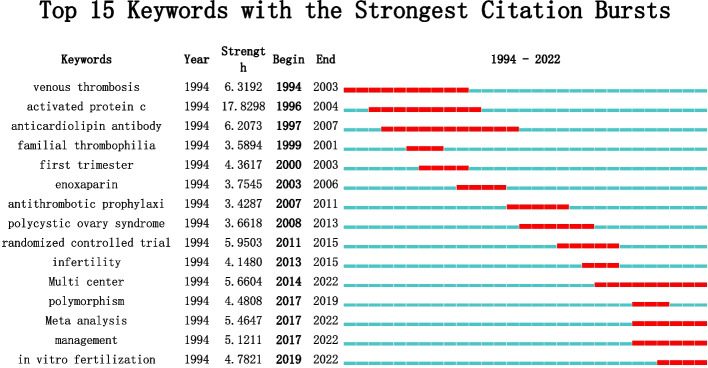
The burst keywords of keywords on thrombophilia with RPL

As shown in Fig. 7, the strongest keyword bursts were analyzed according to the year of publication, which made it possible to find the research hotspots changes over time. We can roughly see the shift from time to time. As can be seen in Fig. 7, there is a long time we focused on the mechanisms and treatment of thrombophilia in relation to RPL, until 2019, researchers began to focus on the impact of thrombophilia in in-vitro-fertilization such as embryo transfer, implantation and growth [32]. The influence of thrombophilia on uterine blood flow and placental trophoblast function exists in both spontaneous pregnancy and IVF [33].

## Discussion

Since the first article about thrombophilia and RPL was published in 1995 [21], 725 articles were published by 3205 authors from 1139 organizations and 68 countries. The knowledge domain and emerging trends in thrombophilia and RPL have been analyzed by scientometric research based on VOSviewer and CiteSpace to offer a better understanding of its development in the past 30 years for researchers.

The annual output of thrombophilia and RPL related publications was steady. The United States kept the lead in publications and citations, followed by Germany and England. The important journals in this area came from these countries such as *thrombosis and haemostasis, human reproduction, lancet,* and *American journal of reproductive immunology.* The *Univ Amsterdam* and *Rambam Med Ctr* were the most important institutions in thrombophilia and RPL with considerable publications and citations. So as the influential authors were from these research teams, such as Brenner from Rambam Med Ctr in Israel, Middeldorp from Univ Amsterdam in the Netherlands. The two institutions combined as two centers in the research of thrombophilia and RPL. Both the research teams and countries needed more communication with each other.

In terms of research propensity, inherited thrombophilia seems attracted more attention than acquired thrombophilia by 2012. As we can see in Fig. 5, Factor V Leiden was the most common genetic risk factor in thrombophilia [34]. Similar genetic factors include prothrombin G20210A (PTGM), methylenetetrahydrofolate reductase (MTHFR) mutations, deficiency of protein S, protein C and antithrombin III (AT3), association of Val34Leu polymorphism of the FXIII (FXIII), 4G/5G polymorphism of plasminogen activator inhibitor (PAI), − 455-G/A polymorphism of β-fibrinogen (fibrinogen), and so on [35, 36]. While there have been so many multiple thrombophilic gene mutations can be detected, there are unknown thrombophilia status of the controls in our research. Some finding showed limited data were available on the association between maternally inherited thrombophilia and RPL [37]. The association between acquired thrombophilia with RPL receive more attention in recent years. Antiphospholipid syndrome (APS) is the only proven thrombophilia that is associated with adverse pregnancy outcome [38]. APS is an autoimmune disease characterized by the presence of antiphospholipid antibodies (aPLs), such as lupus anticoagulant (LA), anticardiolipin antibodies (aCL), and anti-β2-glycoprotein 1 antibodies (β2GPI) [39]. These aPLs are the main laboratory criteria. But the role of classical serological markers is limited in the assessment of the thrombotic risk [27]. And aPLs positive is not the only diagnostic criteria. Therefore, APS is also highly suspected in RPL even lack sufficient evidence. It was widely accepted that aPL caused placental thrombosis, and APLs might change the number of cytokines and hormones and result in inflammation and change in the function of the trophoblast, which led to infertility, RPL, and recurrent implantation failure (RIF) [38, 39]. The application of anticoagulants and immune agents was the research hotspot of APS and RPL. Aspirin or heparin or both for improving pregnancy outcomes in women with persistent antiphospholipid antibodies and RPL [40, 41]. And the antimalarial hydroxychloroquine (HCQ) is currently the center of attention in thrombotic APS [38]. We are looking forward more multi-center randomized controlled trials will be conducted to prove the effectiveness of the treatment.

All in all, even if there are some limitation in currently available laboratory tests, it was still necessary to suggest that the assessment of women with RPL should include the tests associated with the evaluation of thrombophilia [10, 42, 43]. Considering the disruption of uterine blood flow and placental trophoblast function as a result of thrombophilia, to define the possible cut off value by monitoring uterine blood flow in pre-pregnancy and gestation is a good idea for the improvement of the management and treatment [36]. More clinical evidence is still needed.

## Conclusions

There is no doubt about the clear causal relationship between thrombophilia and RPL. Due to the limitations of current medical research, unknown thrombophilia status of the controls still worth exploring. There could be differences in the clinical relevance of different type of thrombophilia, as well as single and multiple thrombophilic factors. Anticoagulation and immunotherapy are currently the main treatment options. More clinical trials and basic research are expected and we should attach more attention to the whole management of in-vitro fertilization in the future.

## Supplementary Information

**Additional file 1.** Appendix.

## Data Availability

The datasets used and/or analysed during the current study are available from the corresponding author on reasonable request.

## Abbreviations

LA: Lupus anticoagulant
RPL: Recurrent Pregnancy Loss
APS: Antiphospholipid syndrome
PTS: Prethrombotic state
MTHFR: Methylenetetrahydrofolate reductase
aPLs: Antiphospholipid antibodies
aCL: Anticardiolipin antibodies
β2GPI: Anti-β2-glycoprotein 1 antibodies
RIF: Recurrent implantation failure
