# Supplementary figures and images for: Research trends and hotspots of recurrent pregnancy loss with thrombophilia: a bibliometric analysis
Source: BMC Pregnancy Childbirth. 2022 Dec 16;22:944. doi: 10.1186/s12884-022-05210-z (PMC9756660; doi:10.1186/s12884-022-05210-z)

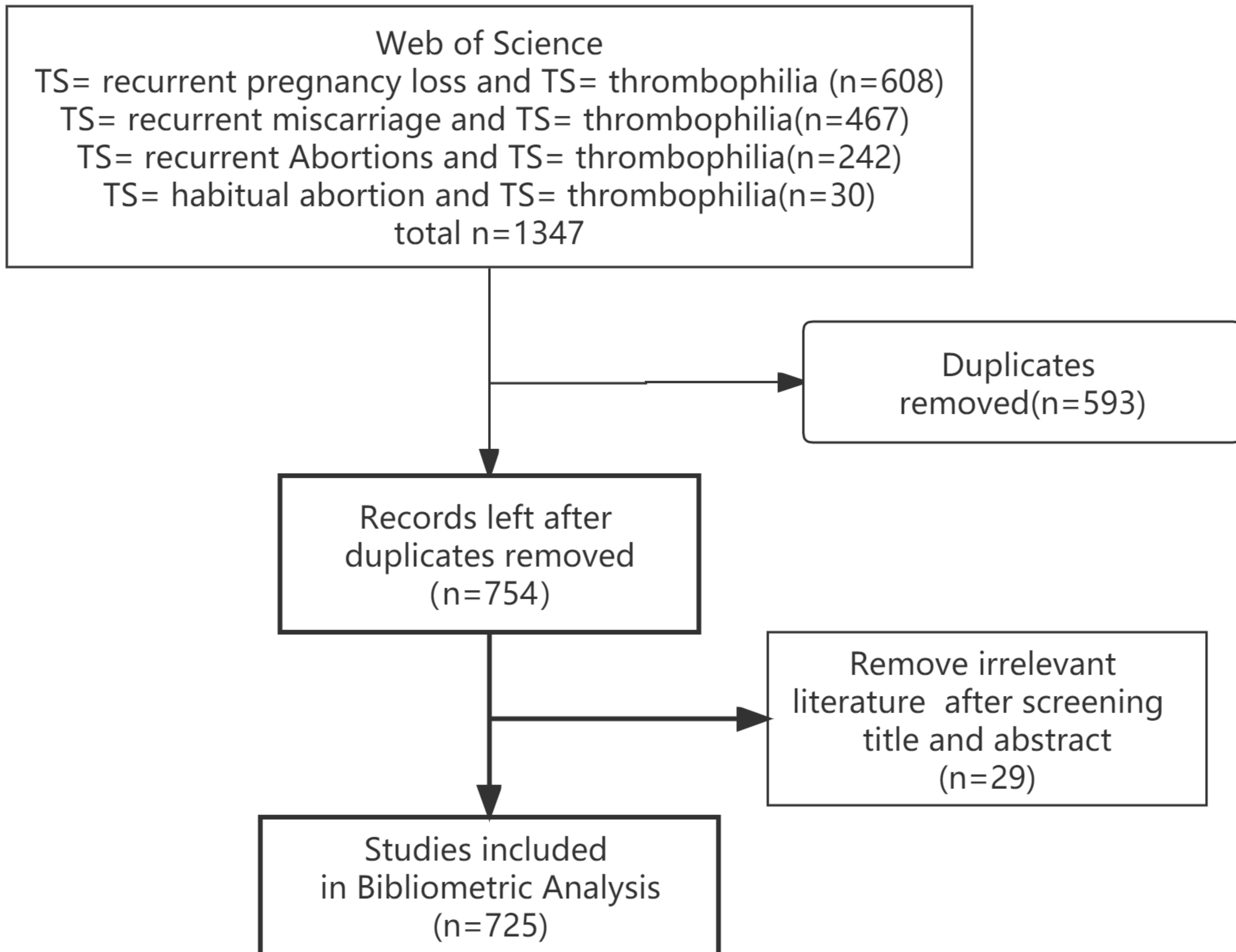

Supplement: Supplementary file 1 — Additional file 1. Appendix. [file 12884_2022_5210_MOESM1_ESM.pdf]
